# Rules Concerning What Patients Tell Experts

**Published:** 1894-12

**Authors:** 


### Rules Concerning What Patients Tell Experts.

The established rules of evidence, it is well known, exclude hearsay testimony. As a consequence, the physician who makes an examination, as an expert, for the purpose of giving testimony in a case, will not, as a general thing, be permitted to state what the patient told him in reference to the specific cause of the injury giving rise to the litigation. But there is another rule, which is just as well settled: the United States Circuit Court of Appeals holds, in the case of the Union Pacific Railway Company v. Novak, decided April 2, 1894, that the physician may testify to what the patient said in describing his bodily condition, and the character and manifestations of his pains, when such statements become necessary to enable the physician to give his opinion as an expert, on account of the latent nature of the facts to be proved by it. The action or non-action of the internal organs can not readily be seen. In such cases the statement of the injured party is ordinarily admissible, in order to enable the expert physician to determine the real nature of the trouble at the time of the examination.—*Journal of the American Medical Association.*

